# Protective CD8 Memory T Cell Responses to Mouse Melanoma Are Generated in the Absence of CD4 T Cell Help

**DOI:** 10.1371/journal.pone.0026491

**Published:** 2011-10-26

**Authors:** Anik L. Côté, Katelyn T. Byrne, Shannon M. Steinberg, Peisheng Zhang, Mary Jo Turk

**Affiliations:** Dartmouth Medical School and the Norris Cotton Cancer Center, Lebanon, New Hampshire, United States of America; MRC National Institute for Medical Research, United Kingdom

## Abstract

**Background:**

We have previously demonstrated that temporary depletion of CD4 T cells in mice with progressive B16 melanoma, followed by surgical tumor excision, induces protective memory CD8 T cell responses to melanoma/melanocyte antigens. We also showed that persistence of these CD8 T cells is supported, in an antigen-dependent fashion, by concurrent autoimmune melanocyte destruction. Herein we explore the requirement of CD4 T cell help in priming and maintaining this protective CD8 T cell response to melanoma.

**Methodology and Principal Findings:**

To induce melanoma/melanocyte antigen-specific CD8 T cells, B16 tumor bearing mice were depleted of regulatory T cells (T_reg_) by either temporary, or long-term continuous treatment with anti-CD4 (mAb clone GK1.5). Total depletion of CD4 T cells led to significant priming of IFN-γ-producing CD8 T cell responses to TRP-2 and gp100. Surprisingly, treatment with anti-CD25 (mAb clone PC61), to specifically deplete T_reg_ cells while leaving help intact, was ineffective at priming CD8 T cells. Thirty to sixty days after primary tumors were surgically excised, mice completely lacking CD4 T cell help developed autoimmune vitiligo, and maintained antigen-specific memory CD8 T cell responses that were highly effective at producing cytokines (IFN-γ, TNF-α, and IL-2). Mice lacking total CD4 T cell help also mounted protection against re-challenge with B16 melanoma sixty days after primary tumor excision.

**Conclusions and Significance:**

This work establishes that CD4 T cell help is dispensable for the generation of protective memory T cell responses to melanoma. Our findings support further use of CD4 T cell depletion therapy for inducing long-lived immunity to cancer.

## Introduction

A major goal of tumor immunotherapy has been the generation of long-lived, protective CD8 T cell memory. However, because many tumor antigens are self-antigens, multiple hurdles must be overcome before functional T cell memory to tumors can be achieved *in vivo* [1]. Our previous work has demonstrated that temporary depletion of CD4^+^CD25^+^ regulatory T cells (T_reg_) in melanoma tumor-bearing mice drives the priming of melanoma/melanocyte antigen-specific CD8 T cells that develop into protective memory following curative excision of the primary tumor [2], [3]. We more recently showed that these CD8 T cells are maintained in a functional state, as long as 600 days following priming, by melanocyte antigen provided in the context of autoimmune vitiligo [4]. Therefore they represent a non-classical type of antigen-dependent T cell memory [4]. In these studies, regulatory T cells were depleted using an antibody to CD4 (mAb clone GK1.5). This is a potent strategy for eliminating immunosuppressive natural CD4^+^ T_reg_ cells, precursors of induced CD4^+^ T_regs_ cells (e.g. IL-35 producing T cells [5]), and suppressive IL-4 producing CD4 T cells [6], although long-term treatment with anti-CD4 could also impair CD8 T cell memory by the elimination of T cell help.

CD4 T cell help has been shown to be a critical component for generating functional CD8 T cell memory against pathogens [7], [8]. However its role in generating functional memory to tumors is less well understood. In acute infectious disease models, CD4 T cell help has been shown to be necessary during the priming phase [9], [10], the maintenance phase [10], [11], and/or the recall phase [11], [12] of the response, but is completely dispensable in other cases [10]. Furthermore, CD4 T cell help has been shown to be required in persistent infection models, where CD8 T cells receive long-term antigen exposure [13]. In models that require T cell help, the absence of CD4 T cells leads to progressive decline in CD8 T cell population size, as well as a loss in T cell effector function and recall capacity [14], [15]. With regards to tumor-expressed self antigens, CD4 T cell help has been shown to improve primary CD8 T cell responses [16], prevent CD8 T cell tolerance by improving dendritic cell function [17], and support secondary recall responses upon viral vaccine boosting [18]. However, our own studies have shown that memory CD8 T cell responses to melanoma/melanocyte differentiation antigens TRP-2 and gp100 can be generated despite early transient ablation of CD4 helper T cells. In these studies, CD4 helper T cells were present early during priming, and again after surgical tumor excision [2], [3], although the importance of CD4 T cell help for the development and maintenance of functional memory to tumor/self antigens has remained unclear.

The goal of the present studies was to investigate whether CD4 T cell help is required for the generation of protective CD8 T cell memory to melanoma. These studies focus on functional memory that is maintained following surgical excision of a primary tumor [3], [4], as a model of protection against tumor recurrence and metastasis. We report that complete depletion of CD4 T cells throughout the entire priming and maintenance phases of the memory response still promotes T cell priming, the generation of autoimmune vitiligo, the development of antigen-specific CD8 T cell memory, and the maintenance of long-lived tumor protection. These studies demonstrate the dispensable nature of T cell help for the generation of CD8 T cell memory to tumor/self-antigens, and establish CD4 T cell depletion as a potent strategy for inducing lasting immunity to cancer.

## Results

### CD4 T cell help is not required for the priming of antigen-specific CD8 T cell responses to B16 melanoma

**Figure 1:**
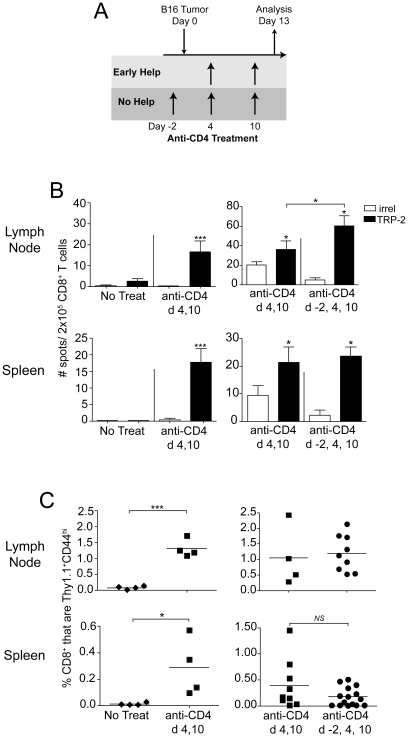
CD4 T cell help is not required for robust primary CD8 T cell responses to B16 melanoma. (A) Schematic diagram of treatment schedules providing either Early Help (light gray) or No Help (dark gray). (B) Mice received primary tumors and anti-CD4 (mAb GK1.5) treatment as indicated in the figure. On day 13, IFN-γ ELISPOT was performed with CD8 T cells isolated from pooled mice (n = 4–9 mice/group) using EL4 cells pulsed with either TRP-2 or irrelevant (OVA) peptides as targets. Data represent average ± SD of four replicate wells. (C) Mice received 10^4^ naïve CD8^+^ Thy1.1^+^ pmel cells one day prior to primary tumor inoculation and anti-CD4 treatment as indicated in the figure. The proportion of Thy1.1^+^ (pmel) cells among total CD8^+^ cells was determined by flow cytometry on day 13. Symbols represent individual mice and horizontal lines represent averages. Statistically significant differences were assessed by *t* test, with * *P*<0.05, *** P<0.0002 and *NS* denoting *P*>0.05. Unless indicated by brackets, asterisks directly above error bars represent significant differences compared with irrelevant (OVA) peptide. Data in (B) are representative of two experiments with similar results; data in (C) are combined from two repeat experiments.

We have previously shown that treatment of mice with anti-CD4 (mAb clone GK1.5) on days 4 and 10 of B16 melanoma tumor growth induces priming of a protective CD8 T cell response against melanoma differentiation antigens, as a result of CD4^+^CD25^+^ T_reg_ depletion [2]. Anti-CD4 treatment did not impair primary tumor growth, although it induced concomitant immunity against re-challenge with melanoma tumors [2]. Non-melanoma tumors grew normally in these mice, demonstrating that tumor-rejection antigens were shared melanoma antigens [2]. Notably, even total depletion of CD4 T cells, beginning 2 days prior to tumor inoculation, induced concomitant tumor immunity in these mice [2]. However, the influence of early T cell help on the priming of melanoma antigen-specific CD8 T cell responses was not assessed. To specifically address this, tumor-bearing mice were treated with anti-CD4 either on days 4 and 10 (early help), or days −2, 4, and 10 (no help), relative to primary tumor inoculation (Figure 1A). Endogenous primary CD8 T cell responses were assessed by IFN-γ ELISPOT on day 12. As we have previously published [2], [3], CD8 T cell responses to TRP-2 were not naturally primed in B16 tumor-bearing mice that did not receive anti-CD4 treatment (Figure 1B). However, CD4 depletion on days 4 and 10 of tumor growth induced significant priming of CD8 T cells specific for TRP-2_180–188_, as compared with an irrelevant peptide control (Figure 1B). Notably, completely eliminating help by CD4 depletion on days −2, 4, and 10, did not impair this CD8 T cell response (Figure 1B). In fact, responses to TRP-2 were significantly greater in hosts that received total CD4 depletion (Figure 1B), which could reflect earlier depletion of CD4^+^CD25^+^ T_reg_ cells in these mice. Because these endogenous T cell responses were small, we also tracked antigen-specific T cell priming by adoptively transferring 10^4^ naïve, congenically (Thy1.1) marked, gp100-specific pmel T cells one day prior to tumor inoculation, as we have previously described [3], [4]. Again, priming of CD8 T cells only occurred in tumor-bearing mice that were treated with anti-CD4 (Figure 1C). In accordance with ELISPOT results, there was no impairment in the priming of pmel cells with an antigen-experienced CD44^hi^ phenotype in mice that had received no help as compared to early help (Figure 1C). Thus, CD8 T cell responses to TRP-2 and gp100 were generated despite the complete absence of CD4 T cell help during priming.

**Figure 2:**
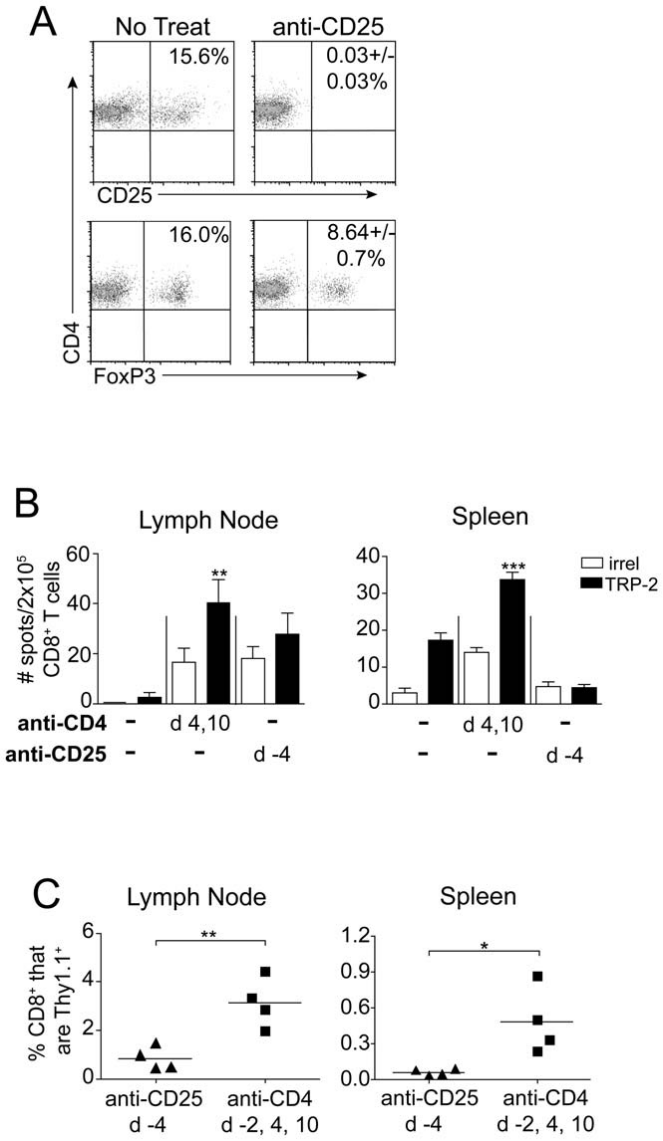
T_reg_ depletion by anti-CD25 treatment fails to prime a CD8 T cell response to B16 melanoma. (A) Mice received anti-CD25 (PC61) treatment, and four days later, the proportion of CD25^+^ and FoxP3^+^ cells among total CD4^+^ cells was determined by flow cytometry. Representative dot plots of 2–6 mice/group are shown; +/− standard deviation. (B) Mice received primary B16 tumors on day 0, and either anti-CD25 or anti-CD4 treatment was given as indicated in the figure. Mice were sacrificed on day 13, and IFN-γ ELISPOT was performed on CD8 T cells (pooled, 4–7 mice/group), with the indicated peptide-pulsed EL4 cells as targets. Data represent average ± SD of four replicate wells. (C) Mice received 10^4^ naïve CD8^+^Thy1.1^+^ pmel cells on day −1, B16 tumors on day 0, and either anti-CD25 or anti-CD4 treatment was given as indicated in the figure. On day 13, the proportion of Thy1.1^+^ pmel cells among total CD8^+^ cells was determined by flow cytometry. Symbols represent individual mice and horizontal lines represent averages. Statistically significant differences were assessed by *t* test, with * *P*<0.05, ** *P*<0.01, *** P<0.001, and *NS* denoting *P*>0.05 as compared with irrelevant peptide (OVA)-pulsed EL4 cells, or as indicated by brackets. Data are representative of two experiments with similar results.

In an attempt to distinguish whether improved CD8 T cell priming could be achieved in the presence of T cell help, mice were alternatively treated with anti-CD25 (mAb clone PC61) to selectively deplete CD25^+^ T_reg_ cells, while preserving CD25^−^CD4^+^ helper T cells. Anti-CD25 treatment was administered once, beginning 4 days prior to primary tumor inoculation, to avoid depletion of effector CD8 T cells, which can also express CD25. Anti-CD25 treatment resulted in total elimination of CD25 expression on CD4 T cells, but only a ∼50% reduction in the Foxp3^+^ CD4 T cell compartment (Figure 2A). Therefore, as previously reported [19], [20], PC61 treatment reduced, but did not completely eliminate, T_reg_ cells. Furthermore, in contrast to anti-CD4, anti-CD25 treatment did not induce detectable priming of TRP-2 specific CD8 T cells in tumor-bearing mice (Figure 2B). Accordingly, significantly smaller populations of pmel cells were detected in anti-CD25 treated mice, as compared with mice lacking total CD4 T cell help (Figure 2C). Therefore, despite its ability to preserve CD4 T cell help, anti-CD25 treatment did not completely deplete T_reg_ cells, nor induce CD8 T cell responses to melanoma. Thus, while our data show that CD8 T cell priming proceeds in the total absence of CD4 T cells (Figure 1B and 1C), the lack of a completely T_reg_-depleted control with fully in tact help precluded a conclusion regarding the potential benefits of help during priming.

### Anti-CD4 treatment is followed by gradual CD4 T cell repopulation, and temporary expansion of polyclonal host CD8 T cells

**Figure 3:**
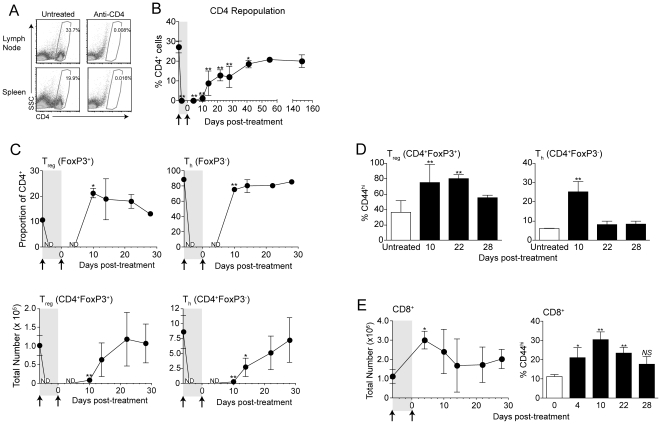
CD4 cells gradually repopulate after anti-CD4 treatment, concurrently with the homeostatic expansion of polyclonal CD8^+^ T cells. (A) Mice received anti-CD4 treatment, and were sacrificed two days later. The proportion of CD4^+^ cells among live cells was determined by flow cytometry. Dot plots represent data from at least 4 mice per group, from 5 total experiments. (B–E) Mice were treated with anti-CD4 on day −6 and day 0, and inguinal lymph nodes were analyzed by flow cytometry at the indicated time points. (B) The proportion of CD4^+^ cells among live cells was determined by flow cytometry. (C) The proportion (top) and total number (bottom) of CD4^+^ cells expressing (left) or lacking (right) FoxP3 expression was determined at indicated time points. ND; not determined (<50 CD4^+^ events were detected). (D) The proportion of CD4^+^FoxP3^+^ (left) or CD4^+^FoxP3^−^ (right) cells expressing CD44 as determined by flow cytometry. (E) The total number of CD8^+^ cells (left) and proportion of CD8^+^ cells expressing CD44 (right) after treatment as determined by flow cytometry. Error bars indicate average ± SD of 3–4 mice per time point. Statistically significant differences were determined by *t* test (C), or one-way ANOVA with Bonferroni post-test (B, D, & E) with * *P*<0.05 and ** *P*<0.01. Data for day 4, 10 and 14 time points were conducted thrice with similar results.

Although often dispensable for primary T cell responses, CD4 T cell help is known to be a critical component of effective memory CD8 T cell responses to viral infections [12], [21]. We have previously demonstrated that CD8 T cells primed by CD4 T cell depletion in B16 tumor-bearing hosts develop into functional memory after surgical tumor excision [3], [4], although details of CD4 T cell repopulation during the period of memory development had not been explored. To assess this, mice were treated with two doses of anti-CD4, 6 days apart, to recapitulate treatment given during priming. As expected, treatment resulted in complete depletion of CD4^+^ cells in lymph nodes within 2 days of the first dose of anti-CD4 (Figure 3A). Similarly, we did not detect significant populations of CD4 T cells in spleens, bone marrow, blood, lungs, or B16 tumors of mice 2–4 days following treatment (data not shown), indicating that depletion was systemic.

Next, CD4 T cell restoration kinetics were assessed. Because our treatment model (Figure 1A) involves B16 tumor removal when CD4 T cells are completely depleted, CD4 T cell repopulation was assessed in tumor-free mice. Beginning 10 days after the second dose of anti-CD4, CD4^+^ T cells became detectable again, with a majority of cells returning by day 28 (Figure 3B). However, CD4^+^ proportions did not reach normal levels until >40 days post-treatment (Figure 3B). We further analyzed differences in repopulation of Foxp3^+^ (T_reg_) and Foxp3^−^ (T_h_) subsets of CD4 T cells following treatment. Repopulation of Foxp3^+^ T_reg_ cells was initially very robust, resulting in T_reg_ populations significantly exceeding normal (undepleted) levels by 10 days following depletion (Figure 3C). Furthermore, consistent with homeostatic proliferation [22], both T_reg_ and T_h_ cells took on an antigen-experienced CD44^hi^ phenotype (Figure 3D). This was more pronounced and sustained in the T_reg_ compartment; affecting >80% of Foxp3^+^ cells at least 22 days post-treatment (Figure 3D). Therefore anti-CD4 treatment transiently eliminated all CD4 T cells, and T_reg_ and T_h_ populations recovered with differing kinetics. Interestingly, CD8 T cells also temporarily expanded to fill the space afforded by CD4 T cell depletion. Four days after anti-CD4 treatment (when CD4 T cells were absent; Figure 3A), total numbers of CD8^+^ cells increased dramatically, with ∼25% of cells taking on a CD44^hi^ phenotype, in accordance with homeostatic expansion (Figure 3E) [22]. This effect was transient, with CD8 T cell numbers returning to normal by ∼14 days post-treatment.

### CD4 T cell help is not required for the generation of protective memory CD8 T cell responses to melanoma

**Figure 4:**
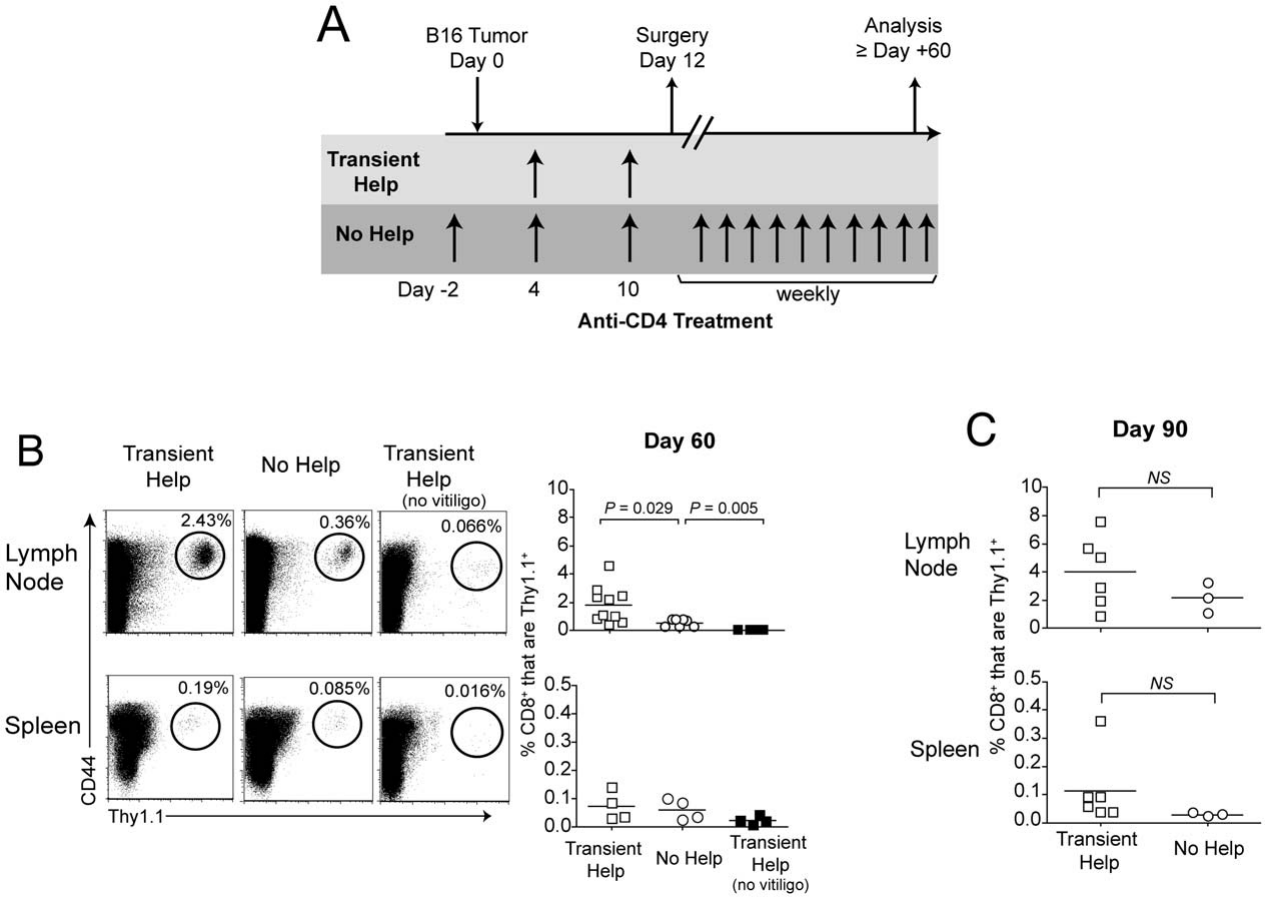
CD4 T cell help enhances, but is not required, for the maintenance of gp100-specific CD8 memory T cells. (A) Schematic diagram of treatment schedule. (B & C) Mice received 10^4^ naïve CD8^+^Thy1.1^+^ (pmel) cells one day prior to treatment as in Panel A, and the proportion of Thy1.1^+^ (pmel) cells among live CD8^+^ cells was determined 60 days (B) or 90 days (C), following surgery. All analyses were performed on vitiligo-affected mice; although unaffected mice were used as a negative control on day 60. Dot plots depicting representative mice are shown. Symbols represent individual mice, and horizontal bars represent averages. Statistically significant differences were assessed by *t* test as indicated by brackets, *NS* denotes *P*>0.05. Data represent 3 combined experiments.

Our data indicated that anti-CD4 treatment on days 4 and 10 of primary tumor growth only transiently eliminated CD4^+^ T cells, with help returning as early as 10 days post-treatment. Therefore, CD4 T cell help could contribute to the development and maintenance of CD8 T cell memory during the post-surgical period [3], [4]. To investigate whether help was crucial for the formation of memory, tumor-bearing mice were treated with anti-CD4 either on days 4 and 10 (transient help); or on days −2, 4, 10, and weekly thereafter (no help). Mice then underwent surgery to excise primary tumors, and enable assessment of memory T cell responses 60 days later (Figure 4A). As previously published [1], [3], [4], we saw no evidence of residual tumor following surgery. In a total of 3 independent experiments, in which mice remained otherwise untreated, only 1 out of 42 tumors recurred following surgery (data not shown).

Because we have recently demonstrated that antigen liberated by autoimmune melanocyte destruction (i.e. vitiligo) supports functional CD8 memory T cell responses after T_reg_ depletion and surgery [4], we first examined whether sustained depletion of CD4 T cells affected the development of vitiligo. We have shown that post-surgical vitiligo is CD8 T cell-mediated, although a role for CD4 T cells was not specifically explored [4]. Indeed, similar proportions of mice developed vitiligo regardless of whether or not CD4 T cell help was present (Table 1). The extent and severity of vitiligo was also unaltered by total CD4 T cell depletion (Table 1). Only vitiligo-affected mice were chosen for subsequent analyses, as we have shown that they exclusively maintain protective memory to melanoma [4].

**Table 1:** Total CD4 depletion does not affect vitiligo incidence or severity.

Depigmentation Level	Transient Help Anti-CD4 Days 4, 10	No Help Anti-CD4 Days −2, 4, 10, Weekly thereafter
None	10/63 (15.8%)	11/50 (22%)
Local	17/63 (26.9%)	13/50 (26%)
Systemic	36/63 (57.1%)	26/50 (52%)

Proportions of mice with depigmentation 60 days post-surgery.

Gp100-specific pmel cells were initially used to track memory T cell responses following surgery. On day 60, significantly larger populations of CD44^hi^ pmel cells were detected in lymph nodes of hosts that had received transient help as compared with no help, although this difference was not observed in spleen (Figure 4B). Furthermore, vitiligo-affected mice lacking help still maintained statistically larger populations of pmel memory T cells as compared with negative control vitiligo-unaffected hosts (Figure 4B). Moreover, ninety days post-surgery, pmel populations were not statistically different in hosts that had received transient help vs. no help (Figure 4C). Thus the overall proportion of antigen-specific memory T cells was only marginally and transiently reduced in the total absence of CD4 T cell help.

**Figure 5:**
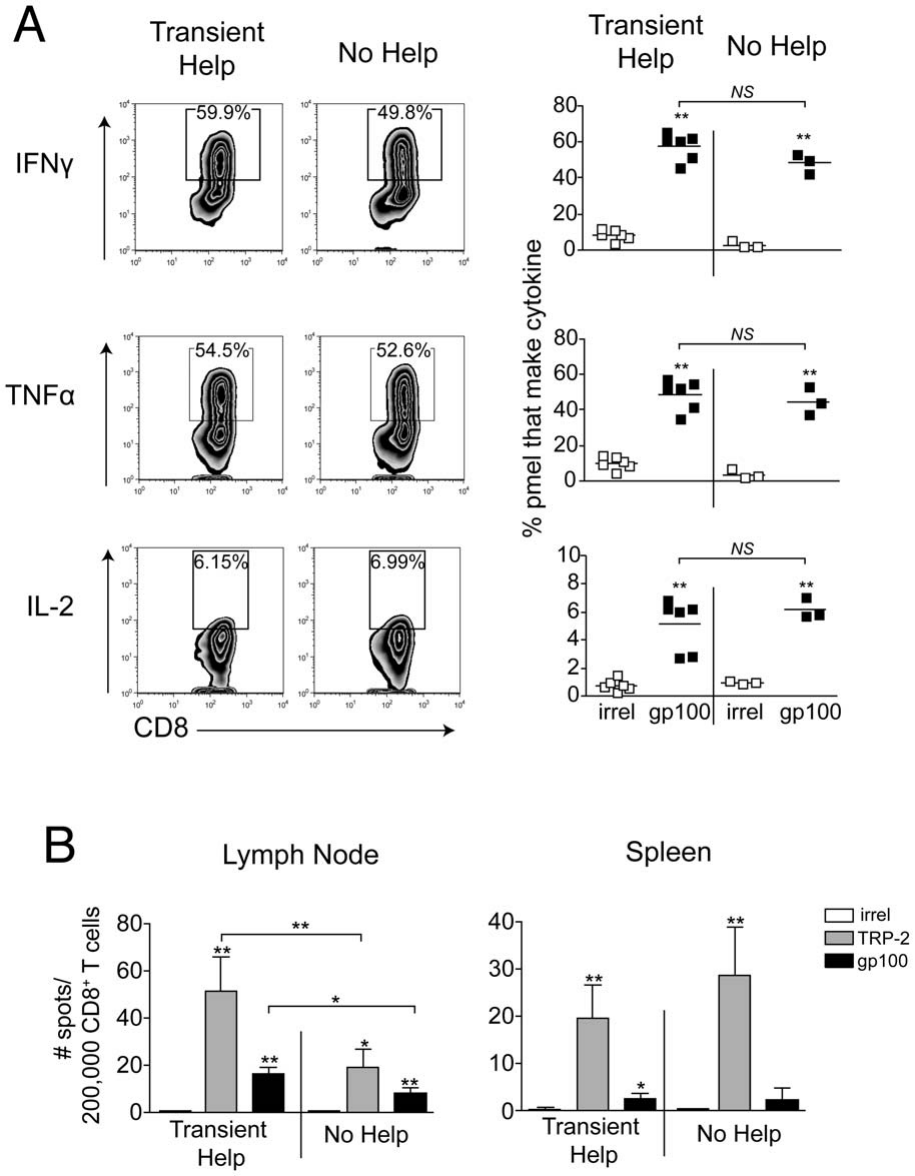
Tumor-specific CD8 memory T cells do not require CD4 T cell help for efficient production of effector cytokines. (A) Mice received 10^4^ naïve CD8^+^Thy1.1^+^ (pmel) cells one day prior to treatment as in Figure 4A. Sixty days following surgery, the proportion of Thy1.1^+^CD8^+^ cells in the lymph node able to produce cytokine upon restimulation with peptide was determined by flow cytometry. Symbols represent individual mice, and horizontal lines indicate averages. Representative contour plots are shown on left. (B) Sixty days following surgery, an IFN-γ ELISPOT was performed with CD8 T cells (pooled, 6 mice/group), using peptide-pulsed EL4 cells as targets. All analyses were performed on vitiligo-affected mice. Bar graphs show 4 replicate wells with errors bars depicting ± SD. Statistically significant differences were determined by *t* test (OVA vs. gp100) or one-way ANOVA with Bonferroni post-test (transient help vs. no help) with * *P*<0.05, ** *P*<0.01, and *NS* denoting *P*>0.05. Data are representative of two experiments with similar results.

Despite normal population sizes, studies have shown that memory CD8 T cells can become functionally impaired in the absence of T cell help [12], [14]. To specifically assess T cell function, pmel cells were restimulated *ex vivo* with gp100 peptide, and cytokine production was measured. We observed that similar proportions of pmel cells produced IFN-γ, TNF-α, and IL-2, regardless of whether or CD4 T cell help had been present during the maintenance phase of the response (Figure 5A). The MFI of cytokine staining was also similar in the presence and absence of help (Figure 5A). Despite this, when endogenous IFN-γ producing memory CD8 T cell were analyzed by ELISPOT, hosts lacking CD4 T cell help demonstrated a significant reduction in responses to TRP-2 and gp100 (Figure 5B). This impairment was observed in lymph nodes but not spleen (Figure 5B), which was similar to the trend observed with pmel cells (Figure 4B). This may suggest that TRP-2-specific CD8 T cells are decreased in number, but not impaired in their capacity to produce cytokine. However, endogenous TRP-2 specific T cell responses were too small for reliable detection by tetramer (not shown), therefore this could not be directly assessed.

**Figure 6:**
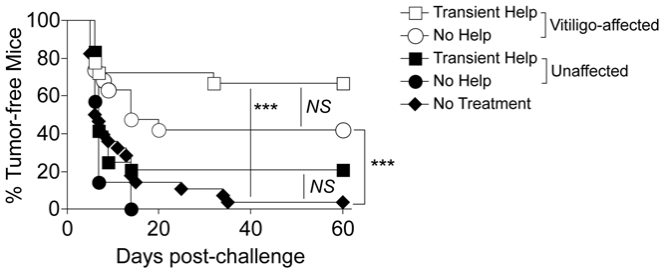
CD4 T cell help is not required for long-lived protection against B16 tumor challenge. Mice were treated as described in Figure 4A. Sixty days post-surgery, mice were stratified based on the development of vitiligo. Vitiligo-affected hosts and unaffected hosts that received either Transient Help or No Help, or naïve mice (No Treatment), were challenged with B16 melanoma cells, and tumor incidence was followed. Comparisons are indicated by brackets, and statistical significance was determined by Log-rank analysis, with *** *P*<0.0002 and *NS* denoting *P*>0.05. Data are combined from 3 experiments, with a total of 8–12 mice per group.

These data showed that functional memory CD8 T cells were generated in the absence of CD4 T cell help, although in slightly reduced proportions. As the ultimate test of T cell memory is the ability to provide long-lived protection, we assessed tumor protection 60 days post-surgery in mice that had received transient help vs. no help. In accordance with our published data [4], mice that did not develop vitiligo after surgery did not maintain long-lived protection against melanoma (Figure 6). This was true regardless of whether these mice received transient help or no help. However, mice with vitiligo that had received transient help demonstrated significant protection against B16 tumor re-challenge (Figure 6). This is consistent with our previous finding that vitiligo-affected mice maintain long-lived CD8 T cell-mediated tumor protection [4]. Importantly, tumor protection was only slightly reduced in mice lacking total help, and this difference did not reach statistical significance (Figure 6). Furthermore, helpless mice demonstrated significant protection as compared with untreated control mice. Therefore CD4 T cell help was not required for protective memory to melanoma.

## Discussion

The generation of protective CD8 T cell memory against tumor-expressed self-antigens has been a major challenge in the field. Our previous work has shown that breaking peripheral tolerance and establishing melanocyte-specific autoimmune disease are two fundamental requirements for generating memory T cell responses to melanoma [3], [4]. The current study now establishes that effective memory CD8 T cell responses against melanoma can be generated even in the complete absence of CD4 T cell help. While we observe minor decreases in the proportion of memory CD8 T cells in lymph nodes of mice lacking help, we find that these cells are not impaired in production of IFN-γ, TNF-α, or IL-2, as has been demonstrated for helpless T cells in viral infection models [12], [14], [15]. Helpless CD8 T cells in our model were also unimpaired in their ability to provide long-lived protection against melanoma re-challenge. Thus while CD4 T cell help could still contribute to the generation of an optimal CD8 T cell response to melanoma, the present studies establish that help is not an absolute requirement for functional, long-lived memory.

This lack of requirement for T cell help appears somewhat contradictory to reports that CD4 help plays a major role in effective CD8 T cell responses to cancer. CD4 helper T cells significantly improve CD8 T cell adoptive therapy of melanoma [23]. OVA specific CD4 T cells have also been show to enhance the expansion of cognate memory CD8 T cells recognizing melanoma-expressed OVA [24]. More recently it has been reported that antigen-specific CD4 helper T cells can enhance recruitment of CD8 T cells to tumors [25], and overcome the immunosuppressive tumor microenvironment [26]. Therefore CD4 T cell help is clearly a critical component of immune responses to cancer.

Specific characteristics of our model may account for our finding that help is not required. First, our studies investigate long-lived memory T cells that develop following surgical excision of a primary tumor. Requirements for T cell help may differ in settings where a large established tumor promotes continual immunosuppression and T cell exhaustion [27], [28]. A second major factor in our studies is the presence of active autoimmune vitiligo, which we have shown to be a fundamental requirement for the maintenance of memory T cell responses to melanoma [4]. Our data show that vitiligo proceeds independently of T cell help. Autoimmunity has not been considered as a variable in other studies, and future work to address stimuli provided by autoimmune vitiligo (e.g. increased antigen load, cytokines, induction of costimulatory molecules on antigen presenting cells, factors released from dying cells), may provide further insight into the unique type of T cell memory that develops in the autoimmune host. Finally, our studies were not without any evidence of a defect within the helpless CD8 T cell compartment. Proportions of gp100-specific T cells were significantly reduced in lymph nodes of helpless mice, 60 days post-surgery. However, our finding that these helpless memory CD8 T cells were unimpaired in their ability to produce cytokines, suggests that hierarchical loss of function followed by deletion, which has previously been documented in viral infection models [14], may not apply to the present setting. It is alternatively possible that the early conversion of effector T cells into memory T cells was impaired in helpless mice. Regardless, reduction in levels of helpless CD8 T cells was unapparent in spleen, insignificant at a later (day 90) time point, and did not result in a significant decrease in tumor protection, and was therefore minor.

Precedence does exist for the generation of functional CD8 T cell memory in the absence of CD4 T cell help. Memory CD8 T cells specific for alloantigen expressed by transplanted skin have been shown to be help-independent [29]. Recent studies in a neu-expressing breast cancer model also demonstrate that the differentiation and recall function of neu-specific CD8 memory T cells are not impaired in the absence of CD4 T cells [30]. These studies provide further support for our findings. Numerous studies now demonstrate the generation of functional anti-tumor immunity as a result of transient CD4 T cell depletion [31], [32], [33], [34]. Thus, in certain cases, the benefits of eliminating multiple suppressive CD4^+^ T cell subsets clearly outweigh the costs of losing CD4 T cell help.

Total CD4 depletion also has unique advantages over other methods of T_reg_ depletion. We found that anti-CD25 treatment was ineffective at priming TRP-2 or gp-100 specific CD8 T cells. This was initially surprising in light of our finding that the same anti-CD25 treatment protocol induces protection against melanoma re-challenge in B16 tumor-excised mice [3]. Indeed different mechanisms of anti-tumor immunity may exist in mice treated with anti-CD4 versus anti-CD25 [31], [35]. Studies by other groups have demonstrated that CD4 T cells and/or NK1.1^+^ cells contribute to tumor protection in B16 tumor-bearing mice treated with anti-CD25 [36], [37], which could explain the absence of detectable CD8 T cell responses. Furthermore, we found that anti-CD25 treatment incompletely depleted T_reg_ cells. In contrast, anti-CD4 treatment efficiently depleted all populations of host CD4 T cells. Our observation that GK1.5 completely depletes the CD4 T cell compartment is supported by recent studies [30], [38], and further contradicts reports of ineffective CD4 T cell depletion [39], [40].

We also found that ablation of the CD4 T cell compartment allowed homeostatic expansion of polyclonal CD8 T cells, which has been shown by other groups to contribute to the preferential recognition of tumor antigens by CD8 T cells [41], [42], [43]. While our data do not directly implicate CD8 T cell homeostatic expansion as a driver of memory CD8 T cell responses in CD4-depleted mice, such expansion may play a minor role. Homeostatic T cell expansion alone, in the absence of a melanocyte antigen-specific immune response, was insufficient for protective memory, as evidenced by a lack of tumor protection in CD4-depleted mice lacking vitiligo. Future studies involving selective T_reg_ depletion in Foxp3-DTR mice should enable better dissection of the importance of T_reg_ depletion vs. CD8 T cell homeostatic expansion. Use of Foxp3-DTR mice in combination with MHC II knockout mice will also provide definitive conclusions regarding the potential contribution of CD4 T cell help in the absence of T_reg_ cells. Targeted depletion of T_reg_ cells in FoxP3-DTR mice has been shown to reduce growth of primary B16 tumors [42], although memory CD8 T cell responses have not yet been assessed in this setting.

In considering anti-CD4 therapy for patients with cancer, the balance between CD4 T cell help and suppression must be considered. Interestingly, studies in patients with metastatic melanoma have revealed overwhelming T_h_2-driven chronic inflammation [44], suggesting that the elimination of CD4 T cells could relieve major barriers to the generation of CD8 T cell immunity in these patients. The present studies support the viability of anti-CD4 therapy in conjunction with surgery for melanoma patients. A humanized anti-CD4 depleting antibody has already demonstrated efficacy in patients with cutaneous T cell lymphoma [45], although its usefulness as immunotherapy against solid tumors remains to be explored. The timing and efficiency of anti-CD4 treatment will be critical factors for consideration, as CD4 T cell repopulation in the presence of antigen, and treatment with non-depleting anti-CD4 antibodies, have been shown to alternatively favor regulatory T cell mediated suppression and transplantation tolerance [46]. Furthermore, TLR-9 agonists and IDO pharmacological inhibitors have been shown to convert T_regs_ into helpers of CD8 T cell responses, suggesting that T_reg_ depletion may not be optimal in certain tumor immunotherapy settings [47].

In summary, the data presented herein show that CD4 T cell help is not required for functional, protective memory T cell responses to cancer. These studies support the concept that memory T cell responses against tumors have a unique set of definitions and requirements that differ from what has been established for foreign pathogens. Additional investigation of antigen-specific CD8 T cell maintenance using poorly immunogenic tumor models will be necessary to further our ongoing understanding of T cell memory to cancer.

## Materials and Methods

### Ethics Statement

This study was carried out in strict accordance with the recommendations in the Guide for the Care and Use of Laboratory Animals of the National Institutes of Health. The protocol was approved by the Institutional Animal Care and Use Committee at Dartmouth College (protocol # 10-09-09). All surgery was performed under isofluorane anesthesia and buprenorphine analgesia, and all efforts were made to minimize suffering.

### Mice and tumor cell lines

C57BL/6 mice (5–6weeks old) were obtained from Charles River Laboratories or The Jackson Laboratory. Pmel-1 (pmel) mice expressing a transgenic TCR specific for gp100_25–33_ in the context of H-2D^b^, on a congenic Thy1.1^+^ background [48], were a gift from Nicholas Restifo (NCI), and were bred and maintained in the specific pathogen-free animal facility at Dartmouth. Male and female mice were used at 6–12 weeks of age.

The B16-F10 (B16) mouse melanoma cell line was originally obtained from Isaiah Fidler (MD Anderson Cancer Center) and passaged intradermally (i.d.) in C57BL/6 mice seven times to ensure reproducible tumor growth. Cell lines were tested by IMPACT and authenticated by the RADIL at the University of Missouri. Tumor cells were cultured in RPMI containing 7.5% FBS and inoculated into mice only if viability exceeded 96%.

### Monoclonal antibodies and peptides

Antibody-producing hybridoma cell lines were obtained from American Type Culture Collection (ATCC). Depleting anti-CD4 (clone GK1.5) and anti-CD25 (clone PC61) were produced as bioreactor supernatants. Antibodies were administered in doses of 250 µg intraperitoneally (i.p). Peptides (>80% purity) were obtained from New England Peptide: TRP-2/DCT_180–188_ (SVYDFFVWL), gp100_25–33_ (EGSRNQDWL), and OVA_257–264_ (SIINFEKL).

### Anti-CD4 treatments in tumor-bearing mice

Tumors were generated by i.d. inoculation of 1.0–1.2×10^5^ live B16 cells. Primary tumors were inoculated in the right flank on day 0, and mice were treated with anti-CD4 mAb clone GK1.5 on days 4 and 10; or on days −2, 4, 10, and continuing weekly until the end of the experiment, as indicated. Tumor diameters were measured thrice-weekly using calipers. Primary tumors were surgically excised from skin, with negative boundaries, on day 12 after tumor cell inoculation. Spontaneous tumor metastasis was not observed with this B16 sub-line, and mice with recurrent primary tumors following surgery (<5%) were removed from the study. Only mice that developed primary tumors (>95%) were used in subsequent analyses.

### IFN-γ Enzyme-linked Immunospot Assay (ELISPOT)

IFN-γ ELISPOT (MabTech) was performed as previously described [2], [49]. Briefly, CD8^+^ T cells from pooled spleens or inguinal lymph nodes were purified using anti-CD8 MACS magnetic beads (Miltenyi Biotec). CD8^+^ T cells were then plated at a 10∶1 ratio with irradiated EL-4 thymoma cell targets (ATCC) that had been pulsed with 1 mg/ml of MHC-I restricted peptide epitopes including TRP-2_180–188_, gp100_25–33_, or OVA_257–264_ (irrelevant peptide) as targets. Cells were incubated for 20 h at 37°C prior to development with aminoethylcarbazole chromogen. Spots were counted using an automated ELISPOT reader system with KS 4.3 software (Karl Zeiss).

### Adoptive transfer and monitoring of pmel T cells

CD8^+^ T cells were magnetically purified using anti-CD8 MACS beads (Miltenyi Biotec) from combined lymph nodes and spleens of 6–8 week old naive Thy1.1^+^ pmel mice, and adoptively transferred at a dose of 10^4^ cells/mouse, one day before primary tumor inoculations. At various time points, mice were euthanized and inguinal lymph nodes and spleens were harvested and mechanically dissociated. Cell suspensions were stained with combinations of the following antibodies: CD8-PerCP (clone 53-6.7; Biolegend), Thy1.1-PE, APC, or PE-Cy7 (clone H1S51; eBioscience), and CD44-FITC, APC, or APC-Cy7 (clone IM7; Biolegend). Flow cytometry was performed on a FACSCalibur or FACSCanto (BD Biosciences), and data were analyzed using FlowJo software (version 8.1, Tree Star).

### Determination of CD4 T cell depletion and repopulation kinetics

Naïve C57Bl/6 mice were injected with 250 µg of GK1.5 anti-CD4 mAb i.p on day −6 and day 0, and were sacrificed at various time points after the first or second injection. Dissociated tissues were assessed for CD4^+^ cell populations by flow cytometry staining with anti-CD4-FITC mAb (clone RM4-4, eBioscience), anti-FoxP3-PE (clone FJK16s; eBioscience), and anti-CD44-APC or APC-Cy7 (clone IM7; Biolegend). CD8 T cell populations were assessed by staining with anti-CD8-PerCP (clone 53-6.7; Biolegend). In mice treated with mAb PC61, samples were stained with anti-CD4 (clone RM4-4, eBioscience), anti-CD25-APC (mAb clone 3C7; BD Biosciences), and anti-FoxP3-PE (clone FJK16s; eBioscience).

### Assessment of autoimmune depigmentation

Vitiligo, observed as the outgrowth of white fur, was assessed sixty days following primary tumor excision, as we have previously described [4].

### Intracellular cytokine staining

Mice received adoptive transfer of 1×10^4^ CD8^+^ pmel cells one day prior to tumor cell inoculation and treatment, as described above. At various time points after surgical tumor excision, lymphocyte samples from spleens and lymph nodes were aliquoted into 96 well plates, and mouse gp100_25–33_ or OVA_257–264_ (irrelevant) peptide was added to a final concentration of 1 µg/ml. IL-2 (10 U/ml) and Brefeldin A (10 µg/ml) were added immediately, and cells were incubated for 5 hours at 37°C. Following incubation, cells were washed and stained with antibodies against CD8 and Thy1.1, and then fixed, permeabilized, and stained intracellularly with the following antibodies: IFN-γ-PE (clone XMG1.2; BioLegend), IL-2-APC (clone JES6-5H4; BioLegend), and TNF-α-FITC (clone MP6-XT22; BioLegend). Flow cytometry was performed as described above.

### Tumor challenge following surgery

1.2×10^5^ live B16 cells were inoculated in the left flank 60 days after surgery. Tumor diameters were measured thrice weekly, and mice were euthanized when tumors reached 10 mm in diameter.

### Statistical analyses

Statistically significant differences between two groups (ELISPOT and flow cytometry) were analyzed by unpaired, Student's two-tailed *t* test, except in the case of intracellular cytokine production where a paired Student's *t* test was used, or experiments with multiple time points where a one-way ANOVA with Bonferroni post-tests was used. For tumor protection experiments, statistical significance was determined by log-rank analysis of Kaplan-Meier data (pooled over strata). Data were considered significant if P≤0.05.
